# The Unfolded Protein Response in Immune Cells as an Emerging Regulator of Neuroinflammation

**DOI:** 10.3389/fnagi.2021.682633

**Published:** 2021-06-11

**Authors:** Dominique Fernández, Antonia Geisse, Jose Ignacio Bernales, Alonso Lira, Fabiola Osorio

**Affiliations:** Laboratory of Immunology and Cellular Stress, Program of Immunology, Institute of Biomedical Sciences, Faculty of Medicine, Universidad de Chile, Santiago, Chile

**Keywords:** UPR, neurodegeneration, microglia, inflammation, neuroinflammation, protein misfolding, ER stress, immune system

## Abstract

Immune surveillance is an essential process that safeguards the homeostasis of a healthy brain. Among the increasing diversity of immune cells present in the central nervous system (CNS), microglia have emerged as a prominent leukocyte subset with key roles in the support of brain function and in the control of neuroinflammation. In fact, impaired microglial function is associated with the development of neurodegenerative diseases, including Alzheimer’s disease (AD) and Parkinson’s disease (PD). Interestingly, these pathologies are also typified by protein aggregation and proteostasis dysfunction at the level of the endoplasmic reticulum (ER). These processes trigger activation of the unfolded protein response (UPR), which is a conserved signaling network that maintains the fidelity of the cellular proteome. Remarkably, beyond its role in protein folding, the UPR has also emerged as a key regulator of the development and function of immune cells. However, despite this evidence, the contribution of the UPR to immune cell homeostasis, immune surveillance, and neuro-inflammatory processes remains largely unexplored. In this review, we discuss the potential contribution of the UPR in brain-associated immune cells in the context of neurodegenerative diseases.

## Introduction

Protein homeostasis, also known as “proteostasis,” is a set of coordinated processes that govern synthesis, quality, control and localization of cellular proteins. Up to a third of protein biosynthesis takes place in the endoplasmic reticulum (ER; Brodsky and Skach, 2011) and thereby, cells possess regulatory mechanisms that maintain proteostasis in conditions that overload the folding capacity of the organelle (a process known as “ER stress”).

**Figure 1:**
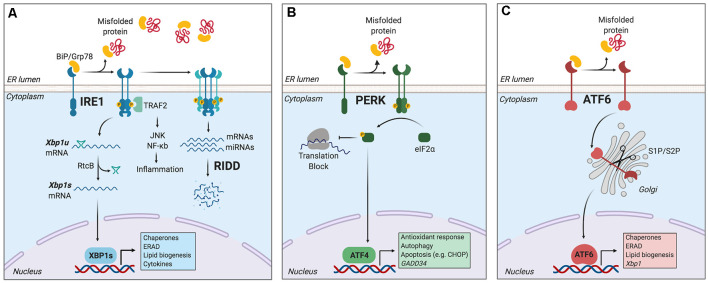
The unfolded protein response (UPR). Endoplasmic reticulum (ER) stress induces an adaptive response known as the unfolded protein response (UPR), which is controlled by three main ER-resident sensors: IRE1, PERK, and activating transcription factor-6 (ATF6). **(A)** IRE1 is activated by oligomerization and trans-phosphorylation upon binding of unfolded proteins and release of the chaperone BiP. IRE1 autophosphorylation leads to the activation of its RNase domain and the processing of the mRNA encoding for X-box binding protein 1 (XBP1s), a transcriptional factor that upregulates genes involved in protein folding and quality control, in addition to regulating ER/Golgi biogenesis and ER-mediated degradation (ERAD), lipid biogenesis and cytokine production. Additionally, IRE1 RNase also degrades a subset of specific RNAs and microRNAs, a process termed Regulated IRE1-Dependent Decay (RIDD). **(B)** Upon activation, PERK phosphorylates the eukaryotic initiation factor-2α (eIF2α), decreasing the synthesis of proteins and the overload of misfolded proteins at the ER. PERK phosphorylation also leads to the specific translation of ATF4, a transcription factor that promotes the expression of genes related to amino acid metabolism, antioxidant response, autophagy, and apoptosis. **(C)** ATF6 is activated upon release of BiP and is translocated to the Golgi, where it undergoes sequential cleavage and removal of its luminal domain. The remaining transactivation domain of ATF6 moves to the nucleus and coordinates the expression of genes encoding ER chaperones, ER-associated protein degradation (ERAD) components, and molecules involved in lipid biogenesis. Figure created with BioRender.com.

The main mechanism counteracting the detrimental effects of ER stress is the unfolded protein response (UPR), a signal transduction pathway that maintains the balance between the folding capacity and the secretory demand of the cell. The UPR is integrated by three ER transmembrane sensors: Inositol-Requiring Enzyme (lRE1), Protein kinase R-like ER Kinase (PERK), and Activating Transcription Factor-6 (ATF6), which are triggered by the accumulation of misfolded proteins in the ER lumen (Figure 1). The UPR transducers act in concert to activate transcription factors and cytosolic signaling modules aiming to restore proteostasis and increase ER biogenesis (Walter and Ron, 2011). Once activated, ATF6 translocates to the Golgi apparatus where it is cleaved by site-1 and site-2 proteases, releasing a transcription factor termed “ATF6-N” that controls expression of ER chaperones, ER-Associated protein degradation (ERAD) components, and lipid biosynthetic genes (Ron and Walter, 2007). PERK, on the other hand, mediates protein translation shutdown *via* phosphorylation of eukaryotic initiation factor-2α (p-eIF2α), which favors selective translation of mRNAs coding for proteins involved in cell survival, ER homeostasis, and antioxidant responses (Kranz et al., 2017). One of these mRNAs encodes ATF4, a transcription factor that controls the expression of the pro-apoptotic factor CHOP (C/EBP homologous protein), and GADD34 (also known as PPP1R15a), a phosphatase 1 cofactor that mediates dephosphorylation of p-eIF2α (Novoa et al., 2001; Hetz and Papa, 2018). Finally, IRE1 is the most conserved sensor of the UPR consisting of a transmembrane protein with two domains: a serine/threonine kinase domain and an endoribonuclease (RNase) domain (Grootjans et al., 2016). The IRE1 RNase domain mediates an unconventional splicing of the mRNA coding for X-box binding protein 1 (XBP1), removing a 26-nucleotide intron, followed by ligation by RtcB ligase (Hetz and Papa, 2018). XBP1 processing results in a shift in the coding reading frame, resulting in translation of the transcription factor “XBP1 spliced” (XBP1s), a master regulator of lipid biosynthesis, ER chaperones, ER biogenesis, and ERAD genes (Lee et al., 2003; Shoulders et al., 2013). Notably, ATF6 and XBP1s can also form heterodimers, amplifying the spectra of proteostatic genes particularly ERAD components (Yamamoto et al., 2007; Shoulders et al., 2013).

Furthermore, in contexts of prolonged ER stress or upon XBP1 deficiency, IRE1 RNase broadens its substrate repertoire and cleaves additional mRNAs/microRNAs through a process termed “Regulated IRE1-Dependent Decay” (RIDD; Lu et al., 2014; Maurel et al., 2014), which regulates mRNAs related to apoptosis and inflammation, among other processes (Maurel et al., 2014). In addition, IRE1 kinase can initiate inflammatory responses through recruitment of TRAF2 (TNF receptor-associated factor 2) protein and the transcription factor NF-κB (Urano et al., 2000; Hu et al., 2006), and it also facilitates apoptosis *via* mobilization of ER Ca^2+^ (Sprooten et al., 2019).

Remarkably, the capacity of the UPR to set the threshold between cell survival and death has associated the pathway with several neurodegenerative diseases that are typified by abnormal protein aggregation (Hetz, 2021). Nevertheless, despite this evidence, it is unclear if the UPR is selectively regulated at the level of supporting cells, nerve cells, and immune cells residing in the brain. In this review, we discuss the potential role of the UPR in controlling neuroinflammation and immunity in the central nervous system (CNS).

## Immune-Surveillance Mechanisms in The CNS

The advent of single-cell analysis has revolutionized our understanding of tissue immunity, demonstrating that the brain contains a broad diversity of immune cell types with roles in homeostasis, aging, and disease (Korin et al., 2017; Mrdjen et al., 2018).

Microglia is the most prominent resident macrophage of the brain parenchyma, where these cells coordinate synaptic pruning, neuron survival/plasticity, and apoptotic cell clearance (Colonna and Butovsky, 2017; Herz et al., 2017). Microglia initiate inflammation through pattern-recognition receptors (PRRs) that recognize noxious stimuli in the CNS (such as protein aggregates, cancer cells, or neurotropic viruses), and the signal for transcription of proinflammatory genes (Colonna and Butovsky, 2017; Prinz et al., 2019), *via* NF-κB or interferon-regulatory factors (IRFs; Reverendo et al., 2019). Relevant PRRs initiating brain inflammation include Toll-like receptors (TLR) such as TLR4 and TLR2, NOD-like receptors (NLRs), and C-type lectins including CLEC7A (Colonna and Butovsky, 2017). Additional mechanisms regulating inflammation are the signaling platforms termed “inflammasomes”, which control the secretion of interleukin-1β (IL-1β) and IL-18 (Swanson et al., 2019). In fact, the inflammasome coordinated by the intracellular sensor NLRP3 (NOD-, LRR- and pyrin domain-containing protein 3) has emerged as a critical regulator of neuroinflammation (Colonna and Butovsky, 2017). As such, microglia adapt to challenges by fine-tuning inflammation, although this response deteriorates with age contributing to neurodegeneration (Scheiblich et al., 2020).

Outside the parenchymal region, there is active immune-surveillance by border-associated macrophages (BAMs), monocytes, T cells, Natural Killer (NK) cells, NKT cells, dendritic cells (DCs), and B cells (Ransohoff and Cardona, 2010; Korin et al., 2017; Mrdjen et al., 2018). BAMs carry out scavenging/patrolling functions, whereas DCs are composed of plasmacytoid DCs (pDCs), type 1 conventional DCs (cDC1s), and type 2 conventional DCs (cDC2s; Mrdjen et al., 2018). pDCs produce type-I interferons (IFN-I) to viral infection, whereas cDC1s and cDC2s elicit long–term immunity *via* antigen presentation to cytotoxic CD8^+^ T cells and CD4^+^ T helper cells, respectively (Mundt et al., 2019; Cabeza-Cabrerizo et al., 2021). T cells in turn protect the brain against antigens found in neurodegenerative diseases and are critical for imprinting the functional maturation of microglia (Pasciuto et al., 2020). Notably, upon inflammation, aging, and neurodegeneration, resident immune cells become activated, and the parenchyma is infiltrated by leukocytes from the periphery, which can perpetuate inflammatory responses and propagate the progression of tissue damage (Scheiblich et al., 2020; Yang et al., 2020). This evidence indicates that the CNS contains a diverse immune microenvironment that can be targeted for intervention of neurodegenerative diseases. Finally, astrocytes are CNS glial cells that also contribute to inflammation (Giovannoni and Quintana, 2020) by expressing PRRs that trigger innate immunity, including TLR4 (Sofroniew, 2020).

Notably, although the UPR is known to control the development and function of immune cells including macrophages, DCs, B cells, eosinophils, NK cells, and T cells in physiological and pathological models (Martinon et al., 2010; Cubillos-Ruiz et al., 2015; Grootjans et al., 2016; Song et al., 2018; Dong et al., 2019), little is known about how the UPR regulates immunity in the CNS. This is a relevant area considering that neurodegenerative diseases including Alzheimer’s disease (AD), Parkinson’s disease (PD), and multiple sclerosis (MS) display altered functions of several immune cell types, many of which are associated with disease progression (Ajami et al., 2018; Mrdjen et al., 2018; Scheiblich et al., 2020). Thus, modulating the UPR in immune cells during the progression of neurodegenerative diseases may have relevant implications for therapy.

## The UPR in Immune Cell Function in Physiological and Inflammatory States

Although most studies connecting the UPR with immunity have focused on organs outside the CNS, some of these findings could be applied to neurodegenerative diseases.

IRE1 *via* XBP1s controls eosinophil, DC, and plasma cell development (Reimold et al., 2001; Iwakoshi et al., 2007; Bettigole et al., 2015), which contribute to neuroinflammation in MS and Experimental Autoimmune Encephalomyelitis (EAE), the mouse model of MS (Greter et al., 2005; Wensky et al., 2005; Mundt et al., 2019; Pröbstel et al., 2020). Furthermore, DCs and plasma cells show constitutive UPR activation and display an elevated protein synthesis rate in steady state (Osorio et al., 2014; Khalsa et al., 2019; Mendes et al., 2020). In these cell types, XBP1s maintains ER architecture, whereas RIDD controls functional aspects, including antigen cross-presentation and cDC1s survival, or immunoglobulin production by plasma cells (Benhamron et al., 2014; Osorio et al., 2014; Tavernier et al., 2017). PERK signaling is also active in DCs (Mendes et al., 2020), whereas ATF6 remains understudied in immunity. These findings suggest that IRE1 and PERK signaling could be targets in neuroinflammatory pathologies involving DCs and plasma cell function, such as EAE (Greter et al., 2005; Mundt et al., 2019; Pröbstel et al., 2020).

The UPR also regulates inflammation (Bettigole and Glimcher, 2015; Grootjans et al., 2016; Flores-Santibáñez et al., 2019), by mechanisms that could be extended to neurodegeneration (Reverendo et al., 2019). Innate recognition *via* TLRs triggers ER stress in leukocytes and UPR components amplify the inflammatory program elicited by these receptors (Goodall et al., 2010; Martinon et al., 2010). ER stress also licenses macrophages to secrete the proinflammatory factors IL-1β, IL-6, TNF, and iNOS (Rao et al., 2014; Shenderov et al., 2014; Yang F. et al., 2018; Yang et al., 2019). Upon bacterial infection, IRE1/XBP1s promotes optimal production of IL-6 and TNF (Martinon et al., 2010; Qiu et al., 2013) and XBP1s favors the synthesis of IL-23, exacerbating psoriasis-like inflammation (Mogilenko et al., 2019). XBP1s also controls prostaglandin synthesis regulating pain behavior (Chopra et al., 2019). In addition, IRE1 RNase activates the inflammasome through degradation of the microRNA miR-17, which is a destabilizer of an NLRP3 activator termed TXNIP (Thioredoxin-Interacting Protein; Lerner et al., 2012; Bronner et al., 2015; Chen D. et al., 2018). Furthermore, IRE1 RNase regulates IFN-I production in microglia, providing a direct connection between IRE1 signaling in CNS-resident immune cells (Studencka-Turski et al., 2019). In fact, XBP1s can bind to IFNβ enhancer and promoter sequences, augmenting IFNβ production (Zeng et al., 2010; Dias-Teixeira et al., 2016). On the other hand, IRE1 kinase also activates innate immunity *via* NOD1/2 receptors and NF-κB (Keestra-Gounder et al., 2016). As such, IRE1 has emerged as an inflammatory regulator through its RNase and Kinase domains (Janssens et al., 2014).

Given its potential, there is interest in targeting IRE1 through the modulation of interacting regulators. Sigma-1 receptor (Sigmar1) is an ER-resident chaperone that regulates IRE1 function (Hayashi and Su, 2007; Mori et al., 2013), and that can be targeted through high-affinity agonists including fluvoxamine, an antidepressant that prevents IRE1-dependent hyperinflammation in sepsis models (Rosen et al., 2019). Thus, IRE1 can be modulated by administration of repurposed medicines.

Furthermore, PERK regulates pro-inflammatory phenotypes of macrophages by activating JAK2-STAT1 signaling pathways (Yang et al., 2019). PERK also controls IL-23 synthesis *via* CHOP (Goodall et al., 2010), and it promotes IL-6, CCL2, and CCL20 production in astrocytes *via* JAK1-STAT3 (Meares et al., 2014; Sanchez et al., 2019). PERK also promotes IFN-I production in DCs (Mendes et al., 2020), and it controls macrophage/myeloid cell function in atherosclerosis and cancer models (DeVries-Seimon et al., 2005; Erbay et al., 2009; Thevenot et al., 2014; Mohamed et al., 2020).

Although little is known about the role of ATF6 in myeloid cells, the UPR sensor regulates pro-inflammatory cytokine production by Kupffer cells in liver ischemia (Rao et al., 2014; So, 2018) and it enhances the pro-inflammatory effect of TLR4 by enhancing NF-κB signaling in macrophages (Rao et al., 2014).

## Potential Crosstalk Between The UPR in Immune Cells and Neuroinflammation

**Figure 2:**
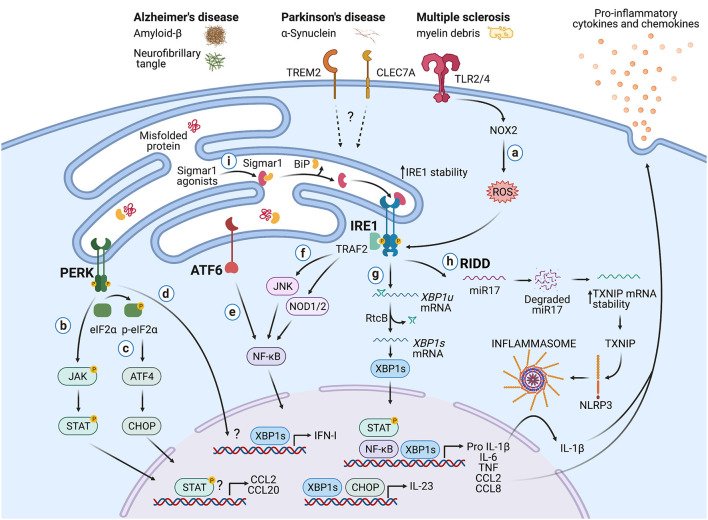
Potential roles of the UPR in immune cells during neurodegeneration. Protein aggregates and myelin debris can promote inflammation *via* triggering of innate receptors and activation of the UPR, which in turn could increase inflammation in neurodegenerative diseases mainly by enhancing the production of proinflammatory cytokines and chemokines. **(a)** Detection of proteins aggregates (Amyloid-β, α-synuclein, neurofibrillary tangles) and myelin debris through TLR2 and TLR4 (and probably others pattern recognition receptors) present on immune cells can activate the IRE1/XBP1s axis through reactive oxygen species (ROS) production by NOX2. **(b)** PERK can modulate the production of the pro-inflammatory cytokines IL-6, TNF, IL-1β, and the chemokines CCL2 and CCL20 through activation of JAK1-STAT3 and JAK2-STAT1 signaling pathways. **(c)** PERK also can control the synthesis of IL-23 *via* CHOP. **(d)** Additionally, PERK could control the synthesis of type I interferons. **(e)** ATF6 *via* NF-κB can enhance the production of the cytokines IL-6, TNF, and the chemokines CCL2 and CCL8. **(f)** The kinase domain of IRE1 can modulate the production of IL-6, TNF, and IL-1β through activation of NF-κB *via* JNK and NOD1/2 receptors. **(g)** BP1s can promote optimal production of IL-6, TNF, and type I IFNs and also favors the synthesis of IL-23. **(h)** IRE1 RNase *via* RIDD activates the NLRP3 inflammasome through degradation of the TXNIP-destabilizing microRNA miR-17, leading to IL-1β production. **(i)** Sigmar1 forms a complex with binding immunoglobulin protein (BiP) under normal conditions, but Sigmar1 agonists can dissociate Sigmar1 from Bip to induce its action as a chaperone protein. Sigmar1 interacts with IRE1 and stabilizes it, prolonging IRE1/XBP1s signaling. Figure created with BioRender.com.

Besides the documented roles in immunity, the UPR is also a hallmark of neurodegenerative diseases (Hetz and Saxena, 2017). Although studies have associated the UPR with the function of neurons and glial cells such as astrocytes and oligodendrocytes (Clayton and Popko, 2016; Godin et al., 2016; Wheeler et al., 2019), the role of the immune-associated UPR during neurodegeneration remains an emerging field. In this section, we highlight potential mechanisms by which the UPR in immune cells could influence CNS pathologies (Figure 2).

Neurodegenerative diseases share protein misfolding, neuronal, loss and inflammation as common mechanisms (Scheiblich et al., 2020). However, evidence demonstrates that AD, PD, and MS are critically regulated by immune-signaling networks, providing a rationale for the study of immunity in these disorders. AD is an age-related disease and common cause of dementia, characterized by a decline in memory and cognitive abilities, neuronal loss, and accumulation of amyloid-β (Aβ) plaques (Holtzman et al., 2011; Efthymiou and Goate, 2017). Mutations in genes coding for amyloid precursor protein and presenilin-1/2 are identified as causes of rare familial AD (Ulland and Colonna, 2018). Nevertheless, most patients with late-onset AD do not carry familial AD mutations and instead, express genetic risk factors associated with immune-related networks, particularly in genes expressed by microglia such as TREM2, CD33, and C1R (Efthymiou and Goate, 2017; Hansen et al., 2018; Schwabe et al., 2020). On the other hand, PD is a progressive disorder characterized by selective degeneration of neuromelanin-containing neurons, especially substantia nigra dopaminergic neurons, and accumulation of α-synuclein (α-syn) aggregates (Zhang et al., 2011). Dopaminergic neuron destruction in PD is connected to genetic, environmental, and immunologic conditions (Koutsilieri et al., 2013). Finally, MS is an inflammatory demyelinating disease displaying three clinical manifestations: a pre-clinical stage; a relapsing-remitting stage, characterized by episodes of neurologic dysfunction and resolution; and a progressive stage (Baecher-Allan et al., 2018). MS pathology is typified by four pathological features: inflammation, demyelination, axonal loss, and gliosis (Constantinescu et al., 2011).

Post-mortem tissue analysis revealed that AD, PD, and MS patients display increased levels of UPR markers including CHOP, BiP, pPERK, p-eIF2α, pIRE1, and XBP1 (Hoozemans et al., 2007, 2009; Mháille et al., 2008; Wheeler et al., 2019). Interestingly, UPR activation in these pathologies is connected to immune cells. Microglia from post-mortem AD patients upregulate transcriptional signatures associated with protein folding and response to unfolded proteins (Mathys et al., 2019). Also, microglia from MS patients show increased expression of BIP, CHOP, and p-eIF2α (Mháille et al., 2008; Cunnea et al., 2011; McMahon et al., 2012), and microglia from animals with EAE also express BiP, GRP94, CHOP, and p-eIF2α (Ní Fhlathartaigh et al., 2013; Ta et al., 2016). Despite this evidence, the relevance of microglial UPR to neurodegeneration remains to be elucidated. In addition, T cells isolated from MS lesions express CHOP (Mháille et al., 2008), whereas spinal cord-infiltrating CD4^+^ T cells from EAE mice display ATF6 activation (Ta et al., 2016).

From an inflammatory perspective, increased levels of IL-6, TNF, and IL-1β are detected in the brain, cerebrospinal fluid, and serum of patients with AD, PD, and MS, although these responses have not been yet linked to IRE1 or PERK activation (Swardfager et al., 2010; Qin et al., 2016; Chen X. et al., 2018; Stampanoni Bassi et al., 2020). Analogous to peripheral macrophages, microglia initiate inflammation upon recognition of Aβ, α-syn, and neuromelanin through TLR2 and TLR4 (Reed-Geaghan et al., 2009; Zhang et al., 2011; Kim et al., 2013). Considering that TLR2 and TLR4 engage the IRE1/XBP1s axis to sustain the production of pro-inflammatory cytokines (Martinon et al., 2010), it is plausible that this UPR branch may initiate neuroinflammation in AD and PD.

Injections of TLR4 agonists are used as a microglial-dependent model of neuroinflammation (Qin et al., 2007; Zhao et al., 2019) and interestingly, this effect can be ameliorated by administration of the pharmacological activator of the UPR, tunicamycin (Wang et al., 2017). It is proposed that mild ER stress induces a reparative phenotype in microglia that protects against neuroinflammation (Wang et al., 2017), and mild ER stress in neurons has also shown to be protective in PD and AD models (Casas-Tinto et al., 2011; Fouillet et al., 2012; Valdés et al., 2014). As such, genetic/pharmacological manipulation of the UPR can have distinct and even opposite effects on neurodegenerative diseases depending on the cell type (Hetz and Saxena, 2017) and thus, it becomes relevant to address the UPR roles in specific cell lineages of the CNS. In fact, the importance of this topic is underscored in studies that show that selective UPR activation *via* the PERK axis in astrocytes results in neuronal degeneration (Smith et al., 2020).

Another regulatory node between the UPR and neurodegeneration is the NLRP3 inflammasome, which is implicated in the pathogenesis of AD, PD, and MS (Song et al., 2017; Zhou et al., 2020). Recognition of Aβ and α-syn activates the NLRP3 inflammasome in microglia (Halle et al., 2008; Codolo et al., 2013; Panicker et al., 2019), and the relevance of this complex is underscored in studies with *Nlrp3*^−/−^ mice, which are protected from spatial memory loss in a model of familial AD (Heneka et al., 2013). Considering that IRE1 regulates the NLRP3 inflammasome and that IRE1 is activated in microglia from AD patients (Lerner et al., 2012; Bronner et al., 2015), it is plausible that the UPR sensor contributes to IL-1β secretion during neurodegeneration.

Perhaps the best-documented link between the UPR and neuroinflammation is in MS (Stone and Lin, 2015). Selective PERK deletion in oligodendrocytes protects against EAE (Lin et al., 2013) and XBP1 deletion in CD4^+^ T cells curtails the differentiation into T helper 17 (Th17) cells, delaying the onset of EAE signs (Brucklacher-Waldert et al., 2017). However, the opposite effect is noticed under ATF4 deficiency, which increases Th17 differentiation and worsens EAE (Yang X. et al., 2018). ATF6 on the other hand is required for transcription of Nos2, Ccl2, and Ccl8 in microglia in this model (Ta et al., 2016). Finally, the IRE1-XBP1s pathway promotes astrocyte-intrinsic pro-inflammatory activities during EAE, which is coordinated by the activity of Sigmar1 (Wheeler et al., 2019). XBP1s promote Nos2 and Csf2 expression in astrocytes and knock-down of Xbp1 in astrocytes ameliorates the disease (Wheeler et al., 2019), providing formal connections between IRE1 activation and CNS inflammation.

## Concluding Remarks

The UPR is a central proteostatic pathway coordinating immunity and inflammation, emerging as a novel therapeutic option for neurodegenerative diseases. Despite the promising advances in the field, the interplay between the UPR and immune cells in the CNS remains to be fully uncovered. Relevant questions should aim to reveal the contribution of UPR transducers and regulators in microglia and additional immune cells contributing to neuroinflammation. Consideration should be given to the aging process, which is regulated by XBP1s in *C. elegans* (Taylor and Dillin, 2013).

## Author Contributions

DF, AG, JB, AL, and FO provided scientific input and wrote the manuscript. All authors contributed to the article and approved the submitted version.

## Conflict of Interest

The authors declare that the research was conducted in the absence of any commercial or financial relationships that could be construed as a potential conflict of interest.
